# Learning Rich Feature Representation and State Change Monitoring for Accurate Animal Target Tracking

**DOI:** 10.3390/ani14060902

**Published:** 2024-03-14

**Authors:** Kuan Yin, Jiangfan Feng, Shaokang Dong

**Affiliations:** 1 School of Computer Science and Technology, Chongqing University of Posts and Telecommunications, Chongqing 400065, China; D230201044@stu.cqupt.edu.cn (K.Y.); D220201004@stu.cqupt.edu.cn (S.D.); 2 College of Artificial Intelligence and Big Data, Chongqing College of Electronic Engineering, Chongqing 401331, China

**Keywords:** animal tracking, deep feature, response map, feature fusion

## Abstract

**Simple Summary:**

Animal movement trajectories are effective indicators of key information such as social behavior, food acquisition, reproduction, migration, and survival strategies in animal behavior analysis. However, manual observation is still relied upon in many analysis scenarios, which is inefficient and error-prone. This paper introduces a computer vision-based method for tracking animal trajectories, which enables monitoring and accurate acquisition of individual target animal movement trajectories over extended periods, overcoming the limitations of manual observation. The experiments demonstrate that the method is efficient and accurate in tracking animals in complex scenes, providing essential basic data for animal behavior analysis and having a wide range of potential applications.

**Abstract:**

Animal tracking is crucial for understanding migration, habitat selection, and behavior patterns. However, challenges in video data acquisition and the unpredictability of animal movements have hindered progress in this field. To address these challenges, we present a novel animal tracking method based on correlation filters. Our approach integrates hand-crafted features, deep features, and temporal context information to learn a rich feature representation of the target animal, enabling effective monitoring and updating of its state. Specifically, we extract hand-crafted histogram of oriented gradient features and deep features from different layers of the animal, creating tailored fusion features that encapsulate both appearance and motion characteristics. By analyzing the response map, we select optimal fusion features based on the oscillation degree. When the target animal’s state changes significantly, we adaptively update the target model using temporal context information and robust feature data from the current frame. This updated model is then used for re-tracking, leading to improved results compared to recent mainstream algorithms, as demonstrated in extensive experiments conducted on our self-constructed animal datasets. By addressing specific challenges in animal tracking, our method offers a promising approach for more effective and accurate animal behavior research.

## 1. Introduction

Animal tracking [1] is a pivotal technology extensively employed in ecology, zoology, and environmental science, and it can help provide insights into wildlife behavior, migration patterns, territory utilization, and interactions within ecosystems. Moreover, it extends into various domains such as resource management, wildlife conservation, and the prediction of disease spread, and these applications provide crucial additional information about the behavior of plants and animals and the health of ecosystems. This technology has progressed significantly over the past few decades, covering many species from small birds to large mammals. Currently, animal tracking technology comprises a spectrum of methods, incorporating radio tracking, satellite tracking, GPS positioning, and approaches based on computer vision. The ongoing advancement of these technologies empowers researchers to systematically and accurately track the movement trajectories of animals, thereby acquiring data pertinent to ecology and physiology.

In recent years, intelligent camera systems have propelled the increasing prominence of computer vision-based animal tracking technology, which collects animal video data by deploying smart cameras and then processes the video data using computer vision methods to track specific animal targets; this process provides foundational data for subsequent analyses of animal behavior. This approach eliminates the need for capturing animals, avoiding the installation of sensors on the animals to collect data. It minimizes interference with the animals to the greatest extent, allowing for data collection in a non-intrusive manner. Moreover, it can collect data for all animals within the camera’s field of view, reducing the cost associated with data acquisition. However, animal tracking technology still faces a number of challenges due to the influences inherent in acquiring video data in unfavorable environmental conditions.

In this paper, we propose a novel computer vision-based animal tracking method (RFCFT) that achieves robust animal trajectory tracking through a correlation filter framework with response map analysis and adaptive feature updating; we utilize the following strategies to achieve high-quality tracking of animals: since animals undergo continuous morphological and scale changes during locomotion, and there are similarities between their appearance and the field environment, a single feature is difficult to represent the animal model robustly, and we constructed two fusion features by simultaneously extracting different layers of deep features and hand-crafted Histogram of Oriented Gradients (HOG) feature in different combinations, which were used to adapt to the complex appearance changes during animal locomotion. In the tracking stage, the two fusion features are used to track the target separately. Subsequently, we analyze the response map of the tracking results and select the tracking outcome with the optimal fusion feature based on the oscillation level of the response map as the predicted target position for the current frame. We analyze the tracking results for each frame and update the model if the results are deemed unreliable. Considering that the motion process of the target varies continuously in the time series, we combine temporal context information with the robust features of the current frame to obtain features for the updated model. Finally, we employ the updated model for re-tracking to yield the ultimate result.

To summarize, the main contributions of this paper are as follows.

We propose an adaptive feature fusion mechanism to construct an optimal animal representation model that blends the semantic information and discriminative advantages of hand-crafted features and multiple layers of deep features to improve the representation of features in complex environments and enhance tracking robustness.

We reformulate the reliability assessment methods. By analyzing the response map, we actively employ two critical indicators within the response map, namely oscillation amplitude and peak variation rate, to comprehensively evaluate the reliability of the tracking results for the current frame, and to promptly monitor the changing state of the target animal.

We have devised a re-tracking model that integrates temporal context information of the target, along with the target’s robust feature and shallow features in the current frame. This model is deployed for re-tracking when the tracking results are deemed unreliable.

Due to the lack of specialized animal tracking datasets, we collected specific animal data from popular datasets. We also obtained partial animal videos from the internet and annotated them ourselves, creating dedicated animal tracking datasets. We further validated our proposed method on this animal dataset, and experimental results confirmed the effectiveness of our tracker.

## 2. Related Works

Traditional visual object tracking methods can be classified into generative and discriminative approaches. However, with the growing interest and development of machine learning techniques in recent years, a variety of machine learning methods are applied in visual object tracking. Examples comprise support vector machines (SVM) [2], correlation filter [3], convolutional neural networks (CNN) [4], recurrent neural networks (RNN) [5], and Siamese neural networks [6]. Hence, the principal research approaches in the visual object tracking field in recent years can be generally divided into two main categories: those based on the correlation filter approach and those using the deep learning approach.

The concept of a correlation filter originated in the field of signal processing. Correlation is an intuitive means to describe the similarity between two variables in signal processing. Leveraging this principle, a filter template is trained using a correlation filter in the context of target tracking. This template is then utilized for executing correlation-based calculations with the target region. Consequently, the current frame’s object position is identified as that corresponding to the maximum output response. Visual object tracking based on correlation filter has yielded significant performance and established itself as one of the most prominent frameworks in recent years.

In 2010, Bolme introduced the MOSSE [3], which paved the way for incorporating correlation filter principles into the visual object tracking domain and exhibited outstanding results. To avoid overfitting problems, CSK [7] introduces regularization terms and kernel methods to convert the linear regression problem into a kernel ridge regression problem [8], which improves the solution of the regression problem and increases the speed of computation. KCF [9] uses HOG [10] features to characterize the target, improving the target representation’s robustness. To estimate the target scale more flexibly, DSST [11] proposes a three-dimensional filter consisting of a one-dimensional scale filter and a two-dimensional position filter to achieve adaptive estimation of the target scale. A cyclic sampling technique is commonly used in correlation filter methods, where training samples are generated by cyclically shifting the original target. However, this practice often leads to boundary effects [12]. To address these boundary effects, SRDCF [13] implements a spatial regularization on the filter that significantly expands the search region, thereby preserving a greater amount of true target information. BACF [14] increases the number of true negative samples by expanding the search to crop small samples, AutoTrack [15] employs local and global information from the response maps to achieve adaptive spatio-temporal regularization. STTCF [16] incorporates a weight matrix into traditional spatial regularization and introduces two long and short-term regularization terms to enhance the performance of the tracker further.

Correlation filter tracking methods combined with deep learning have also been widely developed to improve tracking quality further. HCF [17] proposes to use a pre-trained convolutional neural network framework as a feature extractor, replacing traditional hand-crafted features with deep models that have high-level semantic features. C-COT [18] uses VGG [19] neural network to extract the target features, which effectively improves the robustness of the target features. TCNN [20] utilizes multiple CNN models and builds them into a tree structure for target tracking to enhance the reliability of the model. SINT [6] first proposed a Siamese network-based tracking algorithm, through offline training, to learn a matching function to be utilized for locating the target in subsequent frames. SiamFC [21] adopts the tracking method of offline training and online fine-tuning, which maintains tracking accuracy and improves tracking efficiency.

Benefiting from these works, we adopt correlation filtering as the matching mechanism, but use a different approach to feature selection than traditional correlation filtering tracking algorithms, taking advantage of the different characteristics of hand-crafted features and deep features to complement each other’s strengths, in order to ensure that we can provide a robust representation of a specific individual animal and improve the robustness of tracking. Also different from classical deep learning methods, we extract three deep features from different layers of the VGG network with the fully connected layer removed, and add temporal context information as a supplement when necessary to cope with the complex and variable pose changes of the animal and improve the tracking accuracy.

## 3. Overview

**Figure 1:**
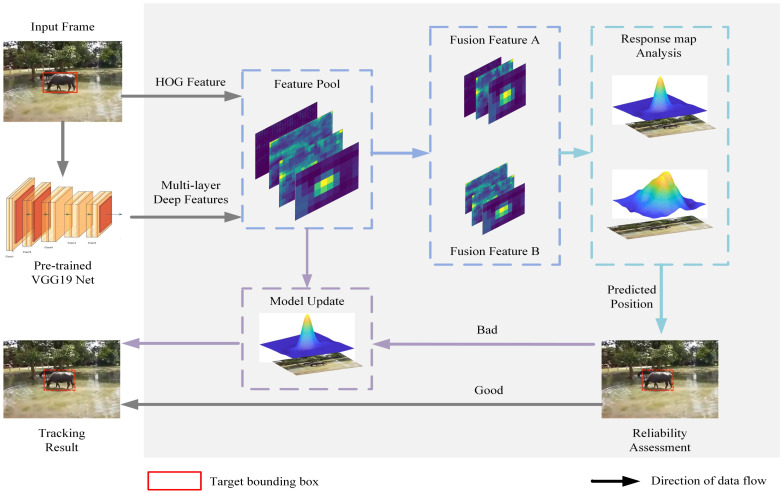
Overview of algorithm.

The algorithm is divided into five parts: (1) use of the labeled data in the first frame of the video sequence to train the correlation filter; (2) input of the target image into the VGG19 network without the fully connected layer, extraction of the deep features of the conv3-4 layer, conv4-4 layer, and conv5-4 layer, and extraction of the hand-crafted feature HOG; (3) use of the combination of full deep features and the combination of deep features and HOG feature to construct two fusion features, then selection of the best fusion features by measuring the average peak-to-correlation energy and the maximum peak of the response map; (4) the optimal fusion feature and the current frame image are input into the correlation filter for correlation calculation, and the predicted position is finally calculated by using the fast Fourier transform; and (5) for unreliable tracking results, updating of the model, building of a new model, and evaluation of the confidence of the updated tracking results to obtain the final tracking results. An overview of the algorithm is shown in Figure 1.

## 4. Method

### 4.1. DCF Framework

The tracking algorithm is based on the DCF framework. DCF is divided into three stages: training, model update, and detection. In the training stage, the filter is trained by a nonlinear regression equation:

(1) $$\min_{w}\sum_{i}∥wx_{i}-y_{i}{∥}_{2}^{2}+\lambda {∥w∥}_{2}^{2}$$

where $x_{i}$ is the data matrix and λ is regularization parameter $\left(\lambda \geq 0\right)$. Using kernel methods to transform a nonlinear space into a linear space and introducing coefficient α to replace filter coefficients w to represent filter solutions within the dual vector space:

(2) $$f\left(x\right)=w^{T}x=\sum_{i}\alpha_{i}k\left(x,x_{i}\right)$$

where $k\left(x,x_{i}\right)$ denotes the kernel function (commonly used kernel functions include Gaussian kernel and polynomial kernel), and $f\left(x\right)$ denotes the response map. The filter can be obtained by the combined solution of (1) and (2):

(3) $$\hat{\alpha }=\frac{\hat{y}}{k^{\hat{x}x}+\lambda^{\prime }}$$

where ^ denotes Fourier transform form in the frequency domain.

In the detection stage, the response map can be obtained using

(4) $$R\left(z\right)=F^{-1}\left({\hat{k}}^{xz}⊙\hat{\alpha }\right)$$

where z denotes the samples in the search region, ⊙ is the element-wise product, ${\hat{k}}^{xy}$ is the result of the kernel correlation operation between the search image block z and the target template *x*, and $F^{-1}\left(\cdot \right)$ is inverse Fourier transform.

In the model update stage, using linear interpolation to update template *x* and the filter coefficients α, calculated by

(5) $${\hat{X}}_{i}=\eta {\hat{X}}_{i}+\left(1-\eta \right){\hat{X}}_{i-1}$$

(6) $${\hat{\alpha }}_{i}=\eta {\hat{\alpha }}_{i}+\left(1-\eta \right){\hat{\alpha }}_{i-1}$$

where η denote learning rate, ${\hat{X}}_{i}$ is the Fourier transform of the cyclic shift sample in i-th frame, ${\hat{\alpha }}_{i}$ is the Fourier transform of the filter coefficient in i-th frame.

### 4.2. Feature Model

**Figure 2:**
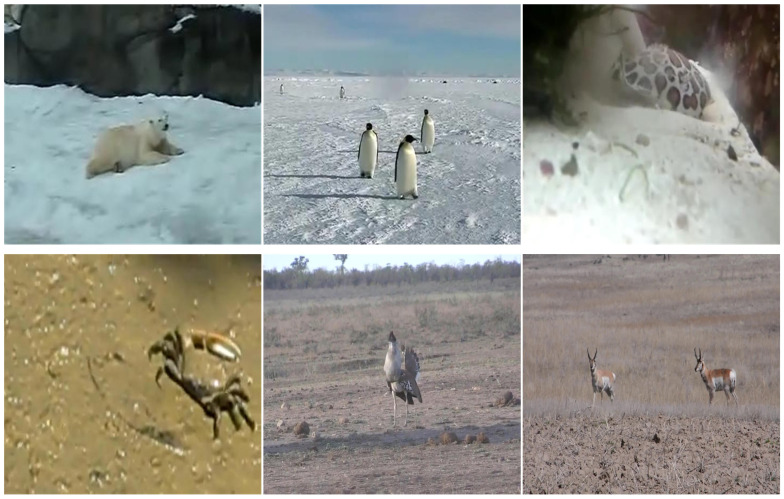
Some wildlife exhibits a significant resemblance in appearance to its surrounding environment.

The continuous evolution of animals results in their colors and forms becoming increasingly similar to their surrounding environment, as shown in Figure 2. Moreover, the complex video capture environment significantly impedes the tracking task. As a result, single features are difficult to model robustly for tracked animals. Consequently, we used a multi-feature fusion approach to construct a feature model of the animal. HOG [10] features exhibit strong illumination invariance and robust resistance to geometric deformations, and HOG partially addresses the significant pose variations in animals and the interferences introduced by the complex field conditions in video images; as the layers of the convolutional neural network become deeper, the deeper features acquire richer semantics, thus enhancing the robustness of the features; nevertheless, this progression comes at the cost of gradually diminishing resolution, impacting the precision of localization. First, we extract the hand-crafted feature HOG, denoted as $F_{HOG}$, then we extract deep features of diverse depths from layers conv3-4, conv4-4, and conv5-4 of the pre-trained VGG19 convolutional neural network model. These are denoted as $F_{CONV3-4}$, $F_{CONV4-4}$, and $F_{CONV5-4}$, respectively. We utilize various feature fusion methods to create two fusion features, consequently enhancing the representational capacity in complex tracking scenarios. The fusion feature is calculated as follows.

(7) $$E_{A}=\omega_{1}\chi_{s}+\omega_{2}\chi_{m}+\omega_{3}\chi_{d}$$

(8) $$E_{B}=\omega_{4}\chi_{h}+\omega_{5}\chi_{m}+\omega_{6}\chi_{d}$$

$F_{CONV3-4}$, $F_{CONV4-4}$ and $F_{CONV5-4}$ are the shallow, medium and deep features extracted from the convolutional neural network, denoted by $\chi_{s}$, $\chi_{m}$, $\chi_{d}$, respectively, $F_{HOG}$ is the hand-crafted feature HOG, denoted by $\chi_{h}$, and $\omega_{i}$ represents the weight parameter.

### 4.3. Reliability Assessment

During tracking, we input the constructed fusion features into a location filter to calculate correlation, thereby obtaining the response map for the current frame. The response map is a Gaussian response map centered on the target, with the peak point on the response map indicating the predicted position of the target in the current frame. Except for the peak, the values of points at other positions in the response map are relatively similar but substantially deviate from the peak value, and a greater peak value corresponds to a more reliable point position. Simultaneously, the overall oscillation level of the response map can also reflect the reliability of the current result, a smaller oscillation level indicates a more reliable tracking result. We utilize both the maximum response peak value $F_{max}$ and the average peak-to-correlation energy (APCE) as integrated discriminative metrics to assess the quality of the tracking results. The calculation method is as follows.

(9) $$APCE=\frac{{\left|F_{max}-F_{min}\right|}^{2}}{mean\left(\sum_{w,h}{\left(F_{w,h}-F_{min}\right)}^{2}\right)}$$

where $F_{max}$ represents the peak value of the response map, $F_{min}$ represents the valley value of the response map, and $F_{w,h}$ represents the corresponding response value at position (w,h). In tracking, we independently compute the $F_{max}$ and APCE values for the two fusion features. When the $F_{max}$ and APCE values calculated for the current frame meet the criteria outlined in Equations (10) and (11), this implies a high degree of reliability in the tracking results for both fusion features. Consequently, the tracking results for the current frame are considered trustworthy, and we select the best outcome from the two fusion features as the final result.

(10) $$F_{max}^{i-th}>\alpha_{1}\times Mean\left(F_{max}\right)$$

(11) $$APCE_{i-th}>\alpha_{2}\times Mean\left(APCE\right)$$

where $F_{\max}^{i-th}$ represents the peak value of the i-th fusion feature, $APCE_{i-th}$ represents the APCE value of the i-th fusion feature, $\alpha_{1}$ and $\alpha_{2}$ are adjustment factors, and $Mean\left(F_{x}\right)$ denotes the mean value of the peaks in the response maps obtained from different fusion features, and $Mean\left(APCE\right)$ denotes the average APCE value of the response maps obtained from different fusion features; herein, the variables $\alpha_{1}$ and $\alpha_{2}$ represent empirical thresholds, with specific values assigned: $\alpha_{1}=0.8$ and $\alpha_{2}$ = 0.8. If the values of $F_{max}$ and APCE do not meet the criteria defined in the formula above, indicating that the tracking results for the current frame lack reliability, the results for the current frame are discarded, and the model update module is initiated.

### 4.4. Model Update

**Figure 3:**
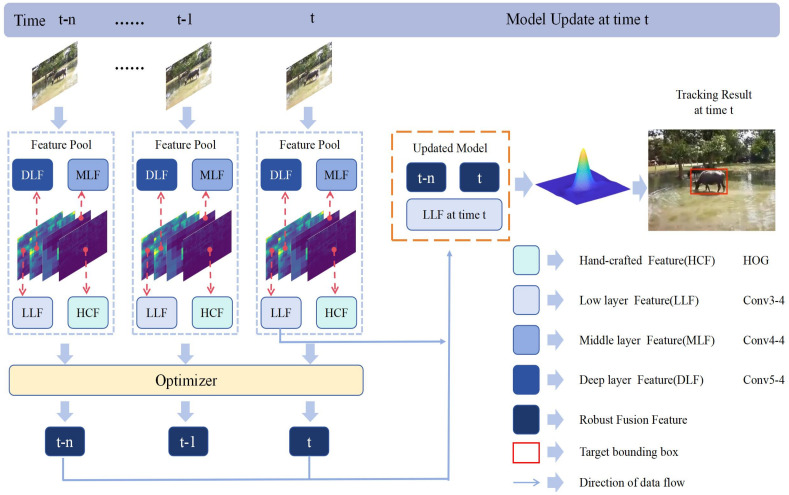
Illustration of model update.

Unreliable tracking results during the tracking process can adversely affect the tracking quality in subsequent frames, and the continuous accumulation of errors in each frame may ultimately lead to tracking failure. Thus, we propose a model update strategy that directly removes unreliable tracking results. Subsequently, employ a new tracking model to retrack the target in the current frame, with the dependable results obtained through this re-tracking process considered as the final target position for the current frame. In light of the continuity of changes in the target over the time series during tracking, we introduce a method to incorporate temporal context information of the target during the re-tracking phase. Integrating the temporal contextual information of the target animal with the robust features of the current frame, along with the shallow features possessing a specific resolution and semantic information in the current frame, collectively constructs the re-tracking feature model for the target animal. The re-tracking feature model is as follows:

(12) $$E_{update}=\omega_{7}\chi_{r-time}+\omega_{8}\chi_{r-current}+\omega_{9}\chi_{s}$$

where $\chi_{r-time}$ is the optimal tracking feature extracted in the case of robust tracking in a short time interval, $\chi_{r-currenr}$ is the optimal fusion feature extracted from the current frame, $\chi_{s}$ is the shallow deep feature of the conv3-3 layer of the current frame, and $\omega_{i}$ is the weight parameter. An overview of the model update is shown in Figure 3.

### 4.5. Algorithm Flow

Combined with the above description of the key steps of our algorithm, the overall process of the algorithm is shown in Algorithm 1.
**Algorithm 1** Proposed Tracking Algorithm**Input:** Initial target position $P_{t-1}=\left(x_{t-1},y_{t-1},w_{t-1},h_{t-1}\right)$, the correlation filter **w****Output:** Estimated object position**Repeat**  1:Crop out the searching window in frame *t* centered at $P_{t}=\left(x_{t},y_{t},w_{t},h_{t}\right)$ and extract HOG features and deep features;  2:Construct two fusion features;  3:For each sample compute response map $R_{z}$ using (4);  4:Estimate the new position $\left(x_{t},y_{t}\right)$ on response map;  5:Estimate the new scale of sample and extract feature;  6:Compute $F_{max}$ and APCE using (9);  7:**if** Not satisfying formula (10) or (11) **then**  8:   Update the model using (12);  9:**end if**   10:Estimate new position $\left(x_{t},y_{t}\right)$ and scale of target;   11:Get the final position $\left(x_{t},y_{t}\right)$ of the current frame;**Until End of video sequence**

## 5. Experiments

### 5.1. Experimental Environment

Our trackers are implemented in MATLAB 2022a on a computer with a 13th Gen Intel(R) Core(TM) i5-13490F 2.50 GHz CPU and 16GB RAM and NVIDIA GeForce 4070 GPU. The MatConvNet toolbox is used to extract the deep features from VGG-19. We use the LaSOT benchmark official tools to test the algorithm and the comparison algorithm in the same environment and analyze the performance of the algorithm using the One-Pass Evolution (OPE) standard; at the same time, seven popular tracking algorithms in recent years are used as a comparison: STRCF [22], DAHCF [23], DSARCF [24], BSTCF [25], AutoTrack [15], ARCF [26], SiamFC [21].

### 5.2. Datasets

**Figure 4:**
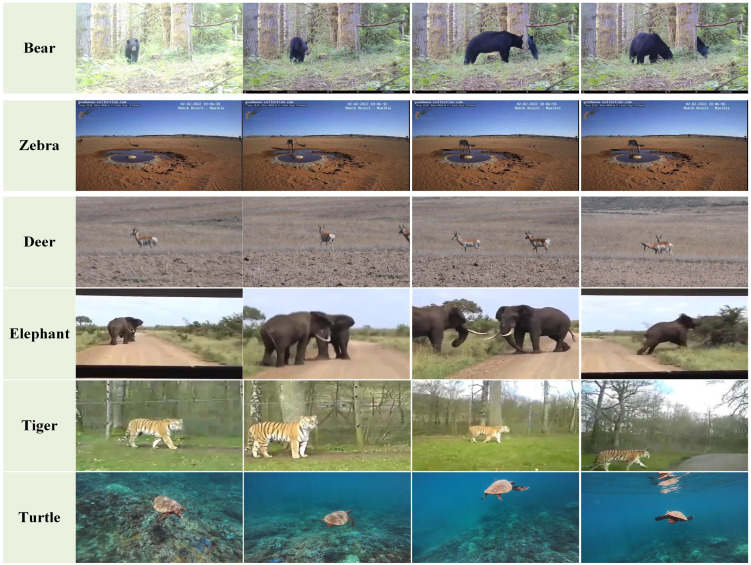
Dataset overviews.

Currently, most public datasets in object tracking primarily focus on tracking generic targets, with a conspicuous absence of specialized datasets for animal tracking. This situation presents a substantial challenge to the progress of animal tracking. To ascertain the robustness of our method, we established a dataset specifically designed for animal tracking. We extracted animal data from existing generic object tracking datasets, including OTB50 [27], OTB100 [28], and LaSOT [29], and supplemented our collection with animal videos obtained from the internet and conducted manual annotations to create our animal datasets. The datasets encompass twenty-nine animal categories, including bears, birds, tigers, elephants, cats, deer, and others. It includes 54 video sequences comprising more than ninety thousand frames. To ensure the effectiveness of the algorithm in real-world scenarios, the datasets we use are captured in authentic environments. The datasets include a broad spectrum of animal video data, spanning various video scenes such as wildlife habitats, domestic settings, public environments, and other scenarios. This contributes to validating the algorithm’s effectiveness in tracking animals across various scenarios. Figure 4 presents a selection of data from the datasets.

To comprehensively evaluate the performance of tracker, as with the LaSOT datasets, we label each sequence with 14 attributes, including out-of-view (OV), partial occlusion (POC), deformation (DEF), motion blur (MB), aspect ratio change (ARC), full occlusion (FOC), fast motion (FM), background clutter (BC), scale variation (SV), rotation (ROT), low resolution (LR), viewpoint change (VC), illumination variation (IV), and camera motion (CM). The attributions are defined in Table 1.

### 5.3. Evaluation Metrics

To objectively evaluate the effectiveness of the algorithm, we utilize precision and success score provided by the OTB tool as evaluation metrics. Precision is defined as the percentage of video frames in which the distance between the estimated center point of the target bounding box generated by the tracking algorithm and the ground-truth center point is less than a specified threshold. In our paper, the threshold is set at 20 pixels. The success score is obtained by calculating the overlap score (OS). If the OS for a specific frame exceeds the designated threshold, we consider that frame as successfully tracked. The overall success score is the percentage of frames identified as successful out of all frames. In our paper, the threshold is set at 0.5, given a tracked bounding box $r_{t}$ and the ground-truth bounding extent $r_{0}$ of a target object, the overlap score is defined as

(13) $$S=\frac{\left|r_{t}\cap t_{0}\right|}{\left|r_{t}\cup t_{0}\right|}$$

where ∩ and ∪ represent the intersection and union operators, respectively, and $\left|\cdot \right|$ denotes the number of pixels in a region.

### 5.4. Experimental Process

**Figure 5:**
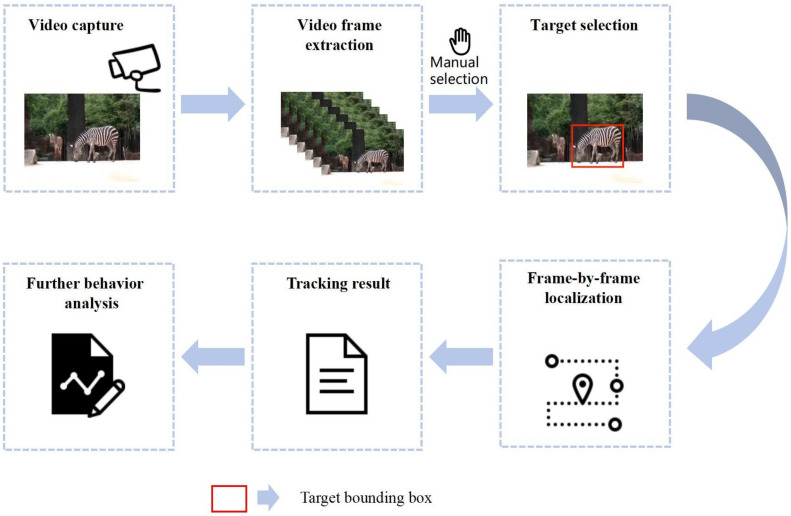
System operation procedure.

In the experiment, all data are derived from real-world scenarios. The experimental procedure is illustrated in Figure 5, and during the operational process, the system simply requires the sequential input of video data frame by frame. In the initial frame, we manually delineated the individuals to be tracked. Subsequently, the system autonomously tracks each frame, extracts position data, and thereby obtains the motion trajectory. Due to the limitations of target detection, which can only identify the category of the target without accurately distinguishing specific individuals within a category, such as a particular deer in a herd; manual selection of the target is necessary when tracking specific individuals. After the initial frame, the system will automatically continue tracking the chosen target.

### 5.5. Ablation Study

To verify the impact of adaptive fusion feature selection and feature update on tracking performance used in this paper, we performed ablation experiments on the datasets. The experimental results are presented in Table 2, where RFCFT-NU represents the algorithm without feature update mechanism, and RFCFT-NF represents the algorithm without adaptive fusion feature selection. It can be observed that, after removing the feature update mechanism, the success rate decreased by 0.5%, and precision decreased by 1.4%. Similarly, without adaptive fusion feature selection, there was a 1.6% decrease in success rate and a 3.2% decrease in precision.

### 5.6. Quantitative Analysis

**Figure 6:**
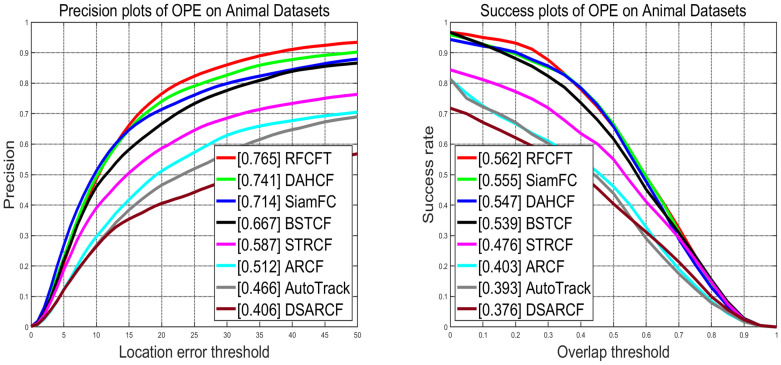
Comparisons with the state-of-the-art tracking algorithms on animal datasets.

For performance evaluation, we compare the proposed RFCFT method with seven other state-of-the-art trackers on our constructed animal datasets. As shown in Figure 6, the proposed method RFCFT achieves the best precision and success scores 76.5% and 56.2%, respectively. However, due to the introduction of animal state monitoring and updating mechanisms in our algorithm compared to traditional algorithms, the complexity of our algorithm has correspondingly increased. Table 3 presents the precision scores, success scores, and fps data for all compared algorithms experimented on the animal dataset, where bold font denotes the optimal values, and underscored values denote the second-best results. Ultimately, our algorithm achieves a speed of approximately 1.915 fps, which still requires further improvement. In terms of algorithm precision, the DAHCF algorithm ranks second with a precision of 74.1%. Compared to DAHCF, our algorithm has improved precision by 2.4% and excels in both real-time performance and success rate. In terms of success rate, the algorithm ranking second is SiamFC, with a success rate of 55.5%. Compared to SiamFC, our algorithm has improved the success rate by 0.7%; despite the lower complexity of the SiamFC algorithm, its accuracy is also 5.1% lower than our algorithm. In overall effectiveness, through comparisons with seven contrasting algorithms, our algorithm achieves the optimal results. Overall, while there is room for improvement in the complexity of our algorithm, it performs exceptionally well in key metrics such as precision and success rate. Through comparison with other contrastive algorithms, our algorithm demonstrates superiority in overall performance.

**Figure 7:**
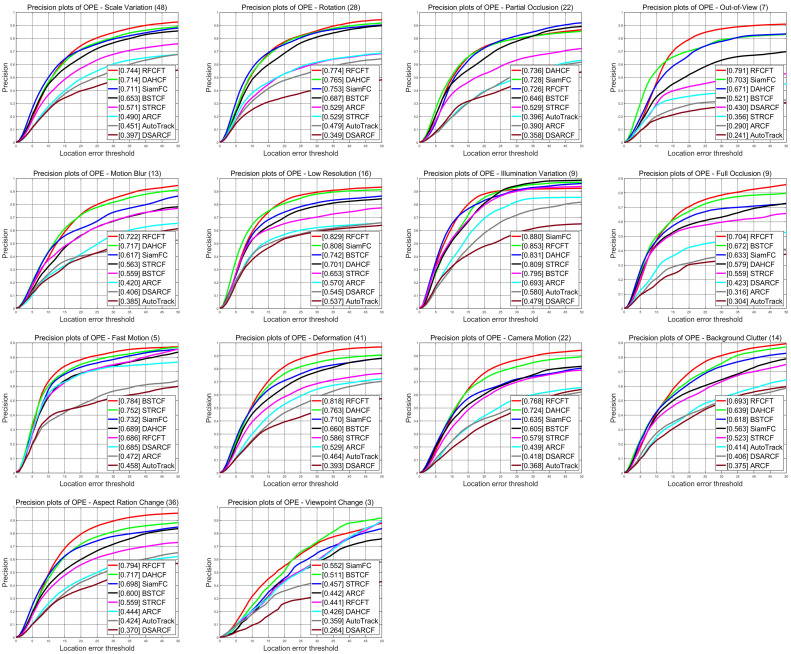
Precision plots evaluated trackers on the animal datasets in terms of 14 challenging attributes.

**Figure 8:**
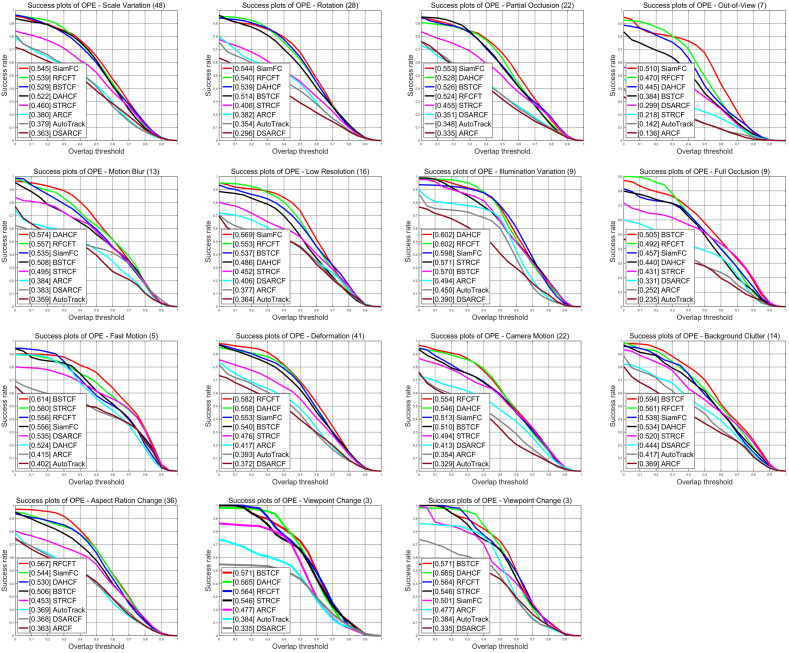
Success plots evaluated trackers on the animal datasets in terms of 14 challenging attributes.

To further validate the capability of our method in handling complex scenarios, we conducted a detailed analysis of the tracking results for different challenging video sequences. Figure 7 shows the tracking precision in various challenging environments, and Table 4 shows the specific values of the precision scores of our tracker and each of the seven other state-of-the-art trackers based on different attributes on the datasets, where the bolded data indicates the optimal data and the underlined data indicates the sub-optimal results. From the results, we can conclude that our method RFCFT has obtained the best results on 10 out of 14 attributes including aspect ratio change (79.4%), background clutter (69.3%), camera motion (76.8%), deformation (81.8%), full occlusion (70.4%), low resolution (82.9%), out-of-view (79.1%), rotation (77.4%), scale variation (74.4%), motion blur (72.2%), and obtained sub-optimal in Illumination Variation. Figure 8 presents the success rate of the tracking process, and we can also see that our method RFCFT has obtained the best results on 3 out of 14 attributes and sub-optimal results on 8 attributes. To conclude, we can demonstrate that the proposed method RFCFT is more effective in distinguishing animals from backgrounds and similar objects and achieves excellent performance improvement in most of the attributes of animal tracking.

### 5.7. Qualitative Evaluation

**Figure 9:**
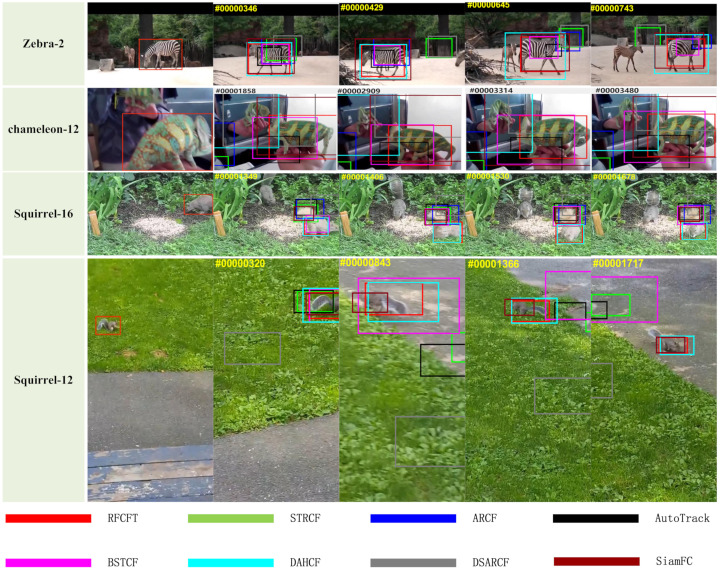
The qualitative results of the algorithm on certain video sequences.

Figure 9 shows some tracking results of our tracker and the other seven trackers on different challenging sequences. As we can see, the tracked zebra in the Zebra2 video sequence suffers from partial occlusion, deformation, rotation, scale variation, and aspect ratio change, initiating from the 345th frame; all other trackers experienced instances of drifting or tracking failures, only our tracker and DAHCF demonstrated the capability to consistently and steadfastly track the target zebra, but the fitting accuracy of our tracker is superior. The tracked chameleon in the chameleon-12 video sequence suffers deformation, background clutter, scale variation, and aspect ratio change; starting from frame 1858, most of the rest of the trackers were confused by the mirrored chameleon in the mirror and experienced tracking failures, and only our tracker was able to track the target stably. In the video sequence squirrel16, the tracked squirrel is notably small, and its color closely resembles the background; additionally, the squirrel appears to fast motion during its trajectory; ultimately, only our tracker and tracker DAHCF successfully tracked the target, most other trackers were confused by the squirrel’s tail, leading to tracking failures. The target squirrel in video sequence squirrel12 suffers from deformation, motion blur, camera motion, rotation, scale variation, out-of-view, low resolution, aspect ratio change and so on; among trackers considered for the analysis, our tracker and SiamFC were able to track the target in most of the frames, all other trackers showed tracking drift, especially DSARCF, which failed to track from frame 320.

## 6. Conclusions

In this paper, we propose an animal tracking algorithm based on correlation filters with response map analysis and adaptive fusion feature update (RFCFT) for tracking specific individual animals’ trajectories. The key contribution is to design a novel adaptive feature fusion mechanism that integrates multi-level deep features and hand-crafted features for effective and efficient animal tracking; by analyzing the response map, we assess the reliability of the tracking results. When the tracking results are unreliable, we introduce temporal information and current frame target information to re-model the target and perform re-tracking to obtain the optimal predicted position; the proposed method automatically enhances the discrimination and suppresses misleading information, enabling online tracking adaptation. In addition, we constructed a specialized animal tracking dataset that enables us to evaluate the effectiveness of animal tracking algorithms objectively. The extensive experimental results on animal datasets demonstrate the effectiveness and robustness of our tracker in comparison with the other seven state-of-the-art trackers.

Furthermore, despite significant findings in achieving a comprehensive balance between tracking accuracy and robustness for individual animals, we candidly acknowledge certain limitations in this study. For instance, the increased complexity resulting from the additional inclusion of animal state change detection and updating modules has elevated the algorithm’s complexity, leading to some loss in tracking speed. Nevertheless, we maintain the belief that these preliminary results provide a valuable foundation for further research.

In future research, we aim to further explore and improve animal trajectory tracking algorithms to address increasingly complex and challenging scenarios. Firstly, we will strive to enhance the accuracy and robustness of the algorithms, particularly in complex environments characterized by large numbers of animals and chaotic motion. Secondly, we plan to investigate methods for better handling occlusions, dynamic backgrounds, and other challenges during the tracking process to improve tracking performance. Additionally, we will explore the utilization of cutting-edge technologies such as deep learning to further enhance algorithm performance, reduce algorithm complexity, and achieve faster end-to-end trajectory tracking. Moreover, we will consider applying the algorithms to a wider range of animal species and real-world application scenarios. Lastly, we aim to strengthen collaboration with fields such as biology and ecology to better understand animal behavior and provide more effective technical support for areas such as animal conservation and ecological environment monitoring.

## Figures and Tables

**Table 1:** Descriptions of 14 different attributes in animal datasets.

Attribute	Definition	Attribute	Definition
CM	Abrupt motion of the camera	VC	Viewpoint affects target appearance significantly
ROT	The target rotates in the image	SV	The ratio of bounding box is outside the rage
DEF	The target is deformable during tracking	BC	The background has the similar appearance as the target
FOC	The target is fully occluded in the sequence	MB	The target region is blurred due to target or camera motion
IV	The illumination in the target region changes	ARC	The ratio of bounding box aspect ratio is outside the rage
OV	The target completely leaves the video frame	LR	The target box is smaller than 1000 pixels in at least one frame
POC	The target is partially occluded in the sequence	FM	The motion of the target is larger than the size of its bounding box

**Table 2:** Results of ablation experiments.

	Precision	Success
RFCFT	**0.765**	**0.562**
RFCFT-NU	0.751	0.557
RFCFT-NF	0.733	0.546

The bolded data represents the optimal result for this metric.

**Table 3:** Tracking performance on the animal datasets.

	RFCFT	DAHCF	BSTCF	STRCF	ARCF	AutoTrack	DSARCF	SiamFC
precision	**0.765**	0.741	0.667	0.587	0.512	0.466	0.406	0.714
Success	**0.562**	0.547	0.539	0.476	0.403	0.393	0.376	0.555
Fps	1.915	0.004	17.309	27.655	22.720	60.801	8.896	**73.910**

The bolded data indicates the optimal data and the underlined data indicates the sub-optimal results.

**Table 4:** Performance evaluations of different attributes on animal datasets.

	RFCFT	DAHCF	BSTCF	STRCF	ARCF	AutoTrack	DSARCF	SiamFC
ARC	**0.794**	0.717	0.6	0.559	0.444	0.424	0.37	0.698
BC	**0.693**	0.639	0.618	0.523	0.375	0.414	0.406	0.563
CM	**0.768**	0.724	0.605	0.579	0.439	0.368	0.418	0.635
DEF	**0.818**	0.763	0.66	0.586	0.529	0.464	0.393	0.710
FM	0.686	0.689	**0.784**	0.752	0.472	0.458	0.685	0.732
FOC	**0.704**	0.579	0.672	0.559	0.316	0.304	0.423	0.633
LR	**0.829**	0.701	0.742	0.653	0.570	0.537	0.545	0.808
OV	**0.791**	0.671	0.521	0.356	0.290	0.241	0.430	0.703
POC	0.726	**0.736**	0.646	0.529	0.390	0.396	0.358	0.728
ROT	**0.774**	0.765	0.687	0.529	0.529	0.479	0.349	0.753
SV	**0.744**	0.714	0.653	0.571	0.490	0.451	0.397	0.711
IV	0.853	0.831	0.795	0.809	0.693	0.58	0.479	**0.880**
MB	**0.722**	0.717	0.559	0.563	0.42	0.385	0.406	0.617
VC	0.441	0.426	0.511	0.457	0.442	0.359	0.264	**0.552**

The bolded data indicates the optimal data and the underlined data indicates the sub-optimal results.

## Data Availability

The data will be available from the authors upon request.

## References

[1] Panadeiro V., Rodriguez A., Henry J., Wlodkowic D., Andersson M. (2021). A review of 28 free animal-tracking software applications: Current features and limitations. Lab Anim..

[2] Ning J., Yang J., Jiang S., Zhang L., Yang M.H. Object tracking via dual linear structured svm and explicit feature map. Proceedings of the IEEE Conference on Computer Vision and Pattern Recognition.

[3] Bolme D.S., Beveridge J.R., Draper B.A., Lui Y.M. (2010). Visual object tracking using adaptive correlation filters. Proceedings of the 2010 IEEE Computer Society Conference on Computer Vision and Pattern Recognition.

[4] Nam H., Han B. Learning multi-domain convolutional neural networks for visual tracking. Proceedings of the IEEE Conference on Computer Vision and Pattern Recognition.

[5] Fan H., Ling H. Sanet: Structure-aware network for visual tracking. Proceedings of the IEEE Conference on Computer Vision and Pattern Recognition Workshops.

[6] Tao R., Gavves E., Smeulders A.W. Siamese instance search for tracking. Proceedings of the IEEE Conference on Computer Vision and Pattern Recognition.

[7] Henriques J.F., Caseiro R., Martins P., Batista J. (2012). Exploiting the circulant structure of tracking-by-detection with kernels. Computer Vision–ECCV 2012, Proceedings of the 12th European Conference on Computer Vision, Florence, Italy, 7–13 October 2012.

[8] Vovk V. (2013). Kernel Ridge Regression, In Empirical Inference: Festschrift in Honor of Vladimir N. Vapnik.

[9] Henriques J.F., Caseiro R., Martins P., Batista J. (2014). High-speed tracking with kernelized correlation filters. IEEE Trans. Pattern Anal. Mach. Intell..

[10] Dalal N., Triggs B. (2005). Histograms of oriented gradients for human detection. Proceedings of the 2005 IEEE Computer Society Conference on Computer Vision and Pattern Recognition (CVPR’05).

[11] Danelljan M., Häger G., Khan F., Felsberg M. (2014). Accurate scale estimation for robust visual tracking. Proceedings of the British Machine Vision Conference.

[12] Liu S., Liu D., Srivastava G., Połap D., Woźniak M. (2021). Overview and methods of correlation filter algorithms in object tracking. Complex Intell. Syst..

[13] Danelljan M., Hager G., Shahbaz Khan F., Felsberg M. Learning spatially regularized correlation filters for visual tracking. Proceedings of the IEEE International Conference on Computer Vision.

[14] Kiani Galoogahi H., Fagg A., Lucey S. Learning background-aware correlation filters for visual tracking. Proceedings of the IEEE International Conference on Computer Vision.

[15] Li Y., Fu C., Ding F., Huang Z., Lu G. Autotrack: Towards high-performance visual tracking for uav with automatic spatio-temporal regularization. Proceedings of the IEEE/CVF Conference on Computer Vision and Pattern Recognition.

[16] Zhao J., Wei F., Chen N., Zhou Z. (2022). Spatial and long–short temporal attention correlation filters for visual tracking. IET Image Process..

[17] Ma C., Huang J.B., Yang X., Yang M.H. Hierarchical convolutional features for visual tracking. Proceedings of the 2015 IEEE International Conference on Computer Vision (ICCV).

[18] Danelljan M., Robinson A., Shahbaz Khan F., Felsberg M. (2016). Beyond correlation filters: Learning continuous convolution operators for visual tracking. Computer Vision–ECCV 2016, Proceedings of the 14th European Conference, Amsterdam, The Netherlands, 11–14 October 2016.

[19] Simonyan K., Zisserman A. (2014). Very deep convolutional networks for large-scale image recognition. arXiv.

[20] Nam H., Baek M., Han B. (2016). Modeling and propagating cnns in a tree structure for visual tracking. arXiv.

[21] Bertinetto L., Valmadre J., Henriques J.F., Vedaldi A., Torr P.H.S. Fully-Convolutional Siamese Networks for Object Tracking. Proceedings of the Computer Vision–ECCV 2016 Workshops.

[22] Li F., Tian C., Zuo W., Zhang L., Yang M.H. Learning spatial-temporal regularized correlation filters for visual tracking. Proceedings of the IEEE Conference on Computer Vision and Pattern Recognition.

[23] Zhang J., Liu Y., Liu H., Wang J., Zhang Y. (2022). Distractor-aware visual tracking using hierarchical correlation filters adaptive selection. Appl. Intell..

[24] Feng W., Han R., Guo Q., Zhu J., Wang S. (2019). Dynamic saliency-aware regularization for correlation filter-based object tracking. IEEE Trans. Image Process..

[25] Zhang J., He Y., Feng W., Wang J., Xiong N.N. (2023). Learning background-aware and spatial-temporal regularized correlation filters for visual tracking. Appl. Intell..

[26] Huang Z., Fu C., Li Y., Lin F., Lu P. Learning aberrance repressed correlation filters for real-time uav tracking. Proceedings of the IEEE/CVF International Conference on Computer Vision.

[27] Wu Y., Lim J., Yang M. Online object tracking: A benchmark. Proceedings of the IEEE Conference on Computer Vision and Pattern Recognition.

[28] Wu Y., Lim J., Yang M. (2015). Object tracking benchmark. IEEE Trans. Pattern Anal. Mach. Intell..

[29] Fan H., Lin L., Yang F., Chu P., Deng G., Yu S., Bai H., Xu Y., Liao C., Ling H. Lasot: A high-quality benchmark for large-scale single object tracking. Proceedings of the IEEE/CVF Conference on Computer Vision and Pattern Recognition.

